# DNA Delivery and Genomic Integration into Mammalian Target Cells through Type IV A and B Secretion Systems of Human Pathogens

**DOI:** 10.3389/fmicb.2017.01503

**Published:** 2017-08-22

**Authors:** Dolores L. Guzmán-Herrador, Samuel Steiner, Anabel Alperi, Coral González-Prieto, Craig R. Roy, Matxalen Llosa

**Affiliations:** ^1^Departamento de Biología Molecular, Universidad de Cantabria (UC), Instituto de Biomedicina y Biotecnología de Cantabria (IBBTEC, UC-CSIC-SODERCAN) Santander, Spain; ^2^Department of Microbial Pathogenesis, Boyer Center for Molecular Medicine, Yale University School of Medicine, New Haven CT, United States

**Keywords:** protein secretion, bacterial conjugation, *Legionella pneumophila*, *Coxiella burnetii*, *Bartonella henselae*, conjugative relaxase, intracellular pathogen, gene therapy

## Abstract

We explore the potential of bacterial secretion systems as tools for genomic modification of human cells. We previously showed that foreign DNA can be introduced into human cells through the Type IV A secretion system of the human pathogen *Bartonella henselae*. Moreover, the DNA is delivered covalently attached to the conjugative relaxase TrwC, which promotes its integration into the recipient genome. In this work, we report that this tool can be adapted to other target cells by using different relaxases and secretion systems. The promiscuous relaxase MobA from plasmid RSF1010 can be used to deliver DNA into human cells with higher efficiency than TrwC. MobA also promotes DNA integration, albeit at lower rates than TrwC. Notably, we report that DNA transfer to human cells can also take place through the Type IV secretion system of two intracellular human pathogens, *Legionella pneumophila* and *Coxiella burnetii*, which code for a distantly related Dot/Icm Type IV B secretion system. This suggests that DNA transfer could be an intrinsic ability of this family of secretion systems, expanding the range of target human cells. Further analysis of the DNA transfer process showed that recruitment of MobA by Dot/Icm was dependent on the IcmSW chaperone, which may explain the higher DNA transfer rates obtained. Finally, we observed that the presence of MobA negatively affected the intracellular replication of *C. burnetii*, suggesting an interference with Dot/Icm translocation of virulence factors.

## Introduction

Bacterial Type IV secretion systems (T4SS) selectively deliver macromolecules to other cells or to the extracellular media. An outstanding feature of these secretion systems is their ability to secrete both, protein and DNA molecules, a particularity that distinguishes them from other types of secretion systems. In addition, the secreted substrates can be delivered to either prokaryotic or eukaryotic cells. This plasticity allows T4SS to be involved in bacterial processes as diverse as horizontal DNA transfer or virulence (Christie, 2016).

Bacterial Type IV secretion systems are multiprotein complexes formed by different constitutive elements: a core complex spanning both bacterial membranes, which forms the transport conduit; a pilus-like appendage, whose function as a transport channel is still under debate; a series of cytoplasmic ATPases, which energize the transport process; and elements necessary to recruit and present the substrates to the translocation machine, including chaperones that are variable for each system (Zechner et al., 2012). Within the family of T4SS, two sub-families were described based on sequence homologies: The Type IV A-IV B secretion systems (T4ASS and T4BSS, respectively). The formers are homologous to the prototypical VirB T4SS of *Agrobacterium tumefaciens* and have been characterized extensively, both functionally and structurally (Chandran Darbari and Waksman, 2015). Members of this family form part of conjugative systems of plasmids such as R388 or RP4; others are encoded in the genomes of human pathogens such as *Bartonella henselae (Bh), Brucella melitensi*s or *Helicobacter pylori* among others, and their main role is to inject virulence factors to the target human cell. Similarly, T4BSS members are encoded in conjugative plasmids such as F, and in the chromosomes of human pathogens such as *Legionella pneumophila (Lp)* and *Coxiella burnetii (Cb)*. Research on T4BSS structure and function lags behind T4ASS; however, extensive work has been done regarding the role of T4BSS-delivered effectors within human cell (Hubber and Roy, 2010; Rolando and Buchrieser, 2014; Personnic et al., 2016).

As aforementioned, a distinctive feature of T4SS is their ability to secrete DNA molecules. This is the main molecular function of T4SS belonging to the conjugative machinery of self-transmissible plasmids (Cabezon et al., 2015). In order to secrete DNA, at least two components are essential in addition to the T4SS machinery: an origin of transfer (*oriT*), which is the DNA sequence required in *cis* on a DNA molecule to be transferred, and a conjugative relaxase, which cuts the DNA strand to be transferred at the *oriT*. Many plasmids also encode for accessory nicking proteins, which assist the DNA processing by the relaxase. The DNA is transferred as a single strand covalently attached to the relaxase, which itself is the substrate of the T4SS; the nucleoprotein complex enters the recipient cell, where the relaxase catalyzes the recircularization of the transferred DNA strand (Garcillan-Barcia et al., 2007; Gonzalez-Perez et al., 2007).

Notably, some conjugative relaxases have the ability to catalyze site-specific recombination between two copies of *oriT*. This phenomenon was first described for the R388 relaxase TrwC (Llosa et al., 1994). TrwC acts as a site-specific recombinase on supercoiled substrates containing minimal target sequences (Cesar et al., 2006). This ability is shared by some, but not all, conjugative relaxases, and it is unclear why. MobA, the relaxase of the mobilizable plasmid RSF1010 (virtually identical to plasmid R1162), is able to catalyze *oriT–oriT* recombination on single-stranded substrates but not on supercoiled plasmid substrates (Meyer, 1989). TrwC can also catalyze the integration of the transferred DNA molecule into a target sequence present in the recipient bacterium (Draper et al., 2005); moreover, the protein can catalyze integration into DNA sequences present in the human genome that resemble its natural target, the *oriT* (Agundez et al., 2012), opening the possibility that this relaxase could work as a site-specific integrase in human cells (Gonzalez-Prieto et al., 2013). Recently, we have shown that the relaxase TrwC is active in a human cell after delivery by the T4SS of *Bartonella henselae*, where it can promote the integration of foreign DNA into the human genome, although without site-specificity (Gonzalez-Prieto et al., 2017). The integration rate of the foreign DNA introduced by TrwC was about 100 times higher compared to when it was introduced by the Mob relaxase from *Bartonella* cryptic plasmid pRGB1, or by transfection.

Gene therapy strategies combine methods to introduce DNA into specific human cell types and to promote DNA integration in the human genome for stable expression. Bacteria have previously been used as vectors for DNA delivery into mammalian cells; the process, known as bactofection, is based on the engulfment of bacteria by an eukaryotic cell, which causes bacterial lysis and DNA release (Celec and Gardlik, 2017). We have previously shown that DNA of any origin and length can be introduced into specific human cell types using *B. henselae* as a delivery agent (Fernandez-Gonzalez et al., 2011). In contrast to bactofection, in this case the DNA is secreted by the living bacterium. *B. henselae* encodes a T4ASS named VirB/D4, which translocates effector proteins to the infected human cell, contributing to its virulence (Saenz et al., 2007). We showed that the VirB/D4 T4SS is also capable of translocating relaxase-DNA complexes via a process resembling bacterial conjugation. DNA transfer was dependent on the conjugative elements required to process the DNA in the donor bacterium, which in this case were derived from the conjugative plasmid R388. No DNA transfer occurred in the absence of the relaxase TrwC, and it was severely impaired in the absence of the conjugative coupling protein TrwB. In a parallel work, Schroder et al. (2011) similarly showed DNA transfer through the *B. henselae* VirB/D4 using the Mob relaxase of a natural plasmid of *Bartonella*; in this case, it was necessary to fuse the known T4 recruiting signal (the BID domain) to the relaxase in order to attain efficient DNA transfer. This discovery had interesting biological implications, opening the possibility that pathogens naturally send DNA to their host cell, and potential biotechnological applications, constituting a new way of DNA delivery to specific human cells (Llosa et al., 2012).

In this work, we asked whether this DNA delivery system could be extended to T4SS from other human pathogens targeting different cell types. We infect cultured mammalian cell lines with *B. henselae, L. pneumophila*, or *C. burnetii*, all containing mobilizable plasmids with markers for eukaryotic selection and encoding different conjugative relaxases. We report that DNA can be delivered to human cells through the T4BSS of *L. pneumophila* and *C. burnetii*, which belong to a distant family of T4SS. This suggests that DNA transfer may be an intrinsic feature of T4SS. DNA transfer and integration rates depend on the relaxase used. All these elements could add to the development of useful tools for *in vivo* genetic modification of human cells. In addition, DNA is a trackable substrate which could be used to study the T4 secretion process in the mammalian host.

## Materials and Methods

### Bacterial Strains and Growth Conditions

Bacterial strains used in this work are listed in **Table 1**. *Escherichia coli* (*Ec*) strains DH5α and D1210 were used for DNA manipulations. *B. henselae* strain RSE247, *L. pneumophila* serogroup 1 strain Lp01 (*hsdR, rpsL*; Berger and Isberg, 1993), and *C. burnetii* strain RSA439 Nine Mile phase II (NMII), or derivatives from these strains as indicated, were used for infection of cultured cells.

**Table 1:** Bacterial strains used in this work.

Name	Relevant genotype	Description/comments	Reference
*Escherichia coli*			
D1210	*recA hspR hsdM rpsI lacI^q^*	Sm^R^, LacI^q^ constitutive expression	Sadler et al., 1980
DH5α T1 phage resistant	*F- φ80lacZΔM15 Δ((lacZYA-argF)U169 recA1 endA1 hsdR17(rk-, mk+) phoA supE44 aaa-thi-1 gyrA96 relA1 tonA*	Nx^R^, T1 phage resistant strain	Killmann et al., 1996
*Bartonella henselae*			
RSE247	Sm^R^	Sm^R^ spontaneous mutant of ATCC 49882	Schmid et al., 2004
*Legionella pneumophila*			
Lp02	Lp01 *thyA*	Spontaneous thymidine auxotroph	Berger and Isberg, 1993
Lp03	Lp02 *dotA*	Spontaneous *dotA* mutant	Berger and Isberg, 1993
CR503	Lp01 *ΔicmSΔicmW*		Coers et al., 2000
*Coxiella burnetii*			
RSA439	Wild type	Plaque-purified Nine Mile phase II (NMII) clone 4	Williams et al., 1981
RSA439 *dotA*::Tn	*dotA*::TnA7	Transposon insertion mutant in *dotA* (CBU_1648), Cm^R^, mCherry	Newton et al., 2014
RSA439 intergenic::Tn	intergenic::TnA7	Transposon insertion mutant between *hemD* and CBU_2078, Cm^R^, mCherry; shows intracellular replication comparable to wild type	Newton et al., 2014

*Escherichia coli* strains were grown at 37°C in Luria-Bertani broth, supplemented with agar for growth on plates. *B. henselae* was grown on Columbia blood agar (CBA) plates at 37°C under a 5% CO_2_ atmosphere. *L. pneumophila* strains were grown on charcoal yeast extract (CYE) plates [1% yeast extract, 1% *N*-(2-acetamido)-2-aminoethanesulfonic acid (ACES; pH 6.9), 3.3 mM L-cysteine, 0.33 mM Fe(NO_3_)_3_, 1.5% Bacto agar, 0.2% activated charcoal] at 37°C, supplemented with 100 μg/ml thymidine if required. *C. burnetii* was grown axenically in liquid acidified citrate cysteine medium 2 (ACCM-2) for 6 days or on ACCM-2 agarose for >8 days at 37°C, 5% CO_2_, and 2.5% O_2_ as previously described (Omsland et al., 2011).

For plasmid selection, antibiotics were added at the following final concentrations: ampicillin (Ap), 100 μg/ml; kanamycin monosulfate (Km), 20 μg/ml (*L. pneumophila*), 50 μg/ml (*E. coli, B. henselae*) or 375 μg/ml (*C. burnetii*); streptomycin (Sm), 300 μg/ml (*E. coli*) or 100 μg/ml (*B. henselae, L. pneumophila*); gentamicin sulfate (Gm), 10 μg/ml (*E. coli, B. henselae*) or 5 μg/ml (*L. pneumophila*); chloramphenicol (Cm), 25 μg/ml (*E. coli*) or 3 μg/ml (*C. burnetii*).

### Plasmids and Plasmid Constructions

Bacterial plasmids are listed in **Table 2**. Oligonucleotides used for plasmid constructions are listed in **Table 3**. Plasmids pAA58, pLG03, pLG04, pMTX808, pMTX821, and pMTX822 were constructed by the isothermal assembly method (Gibson et al., 2009) using the HiFi assembly cloning kit (New England Biolabs). Plasmids pLG05 and pLG06 were constructed by standard restriction cloning techniques (Sambrook and Russell, 2001).

**Table 2:** Plasmids used in this work.

Relaxase	Other conjugative elements	Plasmid	Selection markers^1^	Description	Reference
Mob-BID	pBGR *oriT*	pRS130	Km^R^ Neo^R^	pBGR::*mob:BID*+*gfp*+*neo*	(Schroder et al., 2011)
MobA	RSF1010 *oriT mobB mobC*	RSF1010K	Km^R^	RSF1010 *ΔSm KmR*	(Lessl et al., 1993)
MobA	RSF1010 *oriT mobB mobC*	pAA58	Km^R^	RSF1010K::e*gfp*	This work
MobA	RSF1010 *oriT mobB mobC*	pLG04	Km^R^ Hyg^R^	pAA58::*hyg*	This work
TrwC	R388 *oriT trwA trwB*	pHP159	Gm^R^	pBBR6::*oriT trwABC+egfp*	(Fernandez-Gonzalez et al., 2011)
TrwC	R388 *oriT trwA trwB*	pHP161	Gm^R^	pBBR6::*oriT trwABC+egfp*	(Fernandez-Gonzalez et al., 2011)
TrwC	R388 *oriT trwA trwB*	pMTX821	Km^R^	pHP159::*Km ΔGm*	This work
TrwC	R388 *oriT trwA trwB*	pCOR31	Gm^R^ Neo^R^	pHP159::*neo*	(Gonzalez-Prieto et al., 2017)
TrwC	R388 *oriT trwA trwB*	pLG05	Km^R^ Hyg^R^	pMTX821::*hyg*	This work
TrwC-RalF	R388 *oriT trwA trwB*	pAA12	Gm^R^	pHP159::*trwC*-*RalF TS*	(Alperi et al., 2013)
–	RSF1010 *oriT mobB mobC*	pMTX808	Km^R^ Ap^R^	pAA58::*Ap MobA-*	This work
–	RSF1010 *oriT mobB mobC*	pLG03	Km^R^ Ap^R^ Hyg^R^	pMTX808::*hyg*	This work
–	R388 *oriT trwA trwB*	pHP181	Gm^R^	pBBR6::*oriT trwAB+egfp*	(Fernandez-Gonzalez et al., 2011)
–	R388 *oriT trwA trwB*	pMTX822	Km^R^	pHP181::*Km ΔGm*	This work
–	R388 *oriT trwA trwB*	pCOR35	Gm^R^ Neo^R^	pHP181::*neo*	(Gonzalez-Prieto et al., 2017)
–	R388 *oriT trwA trwB*	pLG06	Km^R^Hyg^R^	pMTX822::*hyg*	This work
nr^2^	nr^2^	pMTX708	Ap^R^ Hyg^R^	pTRE2hyg::*Ptac-oriT*	(Gonzalez-Prieto et al., 2017)
nr^2^	nr^2^	pJB-KAN	Km^R^ Ap^R^	Cloning vector	(Omsland et al., 2011)

*^1R,^resistance to Ampicillin (Ap), Gentamycin (Gm), Kanamycin (Km), Hygromycin (Hyg) or Neomycin (Neo). ^2^nr, not relevant.*

**Table 3:** Oligonucleotides used for plasmid constructions.

Plasmid constructed (IA/RC)^1^	Oligonucleotide sequence (5′ to 3′)^2^	Amplified fragment
pLG03, pLG04 (IA)	TCCAGATGTATGCTCTTCTGCTCGGCGCGCC**TTTCGTCTCGAGGCAGTG**	Hyg^R^ cassette
	TGCGATGATAAGCTGTCAAACAGGCGCGCC**GTCAGTTAGGGTGTGGAAAG**	
pLG05, pLG06 (RC)	CCAAACATCGAT**GTCAGTTAGGGTGTGGAAAG**	Hyg^R^ cassette
	CCAAACATCGAT**CTTTCGTCTCGAGGCAGTG**	
pAA58 (IA)	**AGCTTGCCGCCGCCGCAG**	RSF1010K
	**GGTCTATTGCCTCCCGGTATTCCTGT**	
	**CGCCCAGATCATCGACTTACAGGAATAC**	
	**GAGCAGAAGAGCATACATCTGGAAGC**	
	GCCGCTTTCCTGGCTTTGCTTCCAGATGTATGCTCTTCTGCTCGGCGCGCC**TGTTTGACAGCTTATCATCGCAG**	eGFP cassette
	GTGCGGATGAAGTCAGCTCCACCTGCGGCGGCGGCAAGCTCCTGCAGG**CCCCGACACCCGCCAACAC**	
pMTX808 (IA)	GCACCTGACCGGTGCCGAGCGCCTGCCGTATTG**AGAAGGCCATCCTGACGGA**	Ap^R^ cassette
	TCGCCGCCACCGGCATGGATGGCCAGCGTA**TTACCAATGCTTAATCAGTGAG**	
pMTX821, pMTX822 (IA)	AGTATGGGCATCATTCGCACATGAA**GGCGATTCGCCGCTTTC**	Km^R^ cassette
	GGTGGCGGTACTTGGGTCGAT**TTATCAGAAGAACTCGTCAAG**	Km^R^ cassette

*^1^IA, isothermal assembly; RC, restriction cloning. ^2^Nucleotides annealing to the PCR template are shown in bold, and restriction sites used for cloning are underlined.*

pAA58 was generated by assembling the eGFP eukaryotic expression cassette from pHP161 into the PstI sites of RSF1010K, which was itself amplified in two overlapping PCR fragments. To generate pLG03, pLG04, pLG05, and pLG06, the hygromycin resistance cassette from pMTX708 was amplified and assembled into the SgsI site of pMTX808 and pAA58, or into the ClaI site of pMTX821 and pMTX822, respectively. pMTX808 was constructed by insertion of an ampicillin resistance cassette (amplified from pJB-KAN) into the *mobA* gene of pAA58. The cassette was inserted at the unique BstZ17I site which lies at nt 320 of *mobA*, leaving unaffected the downstream *mobB* and *repB* ORFs which overlap *mobA*. pMTX821 and pMTX822 were generated by insertion of a kanamycin resistance cassette from pJB-KAN into the gentamicin resistance cassette of pHP159 and pHP181, respectively.

Plasmids were routinely introduced in all strains by electroporation. The protocol for *C. burnetii* electroporation was previously described (Newton et al., 2014); electroporation was carried out with a Bio-Rad GenePulser Xcell (settings: 1.8 kV, 500 Ω, 25 μF). To make competent *L. pneumophila* cells, bacteria were collected from 48 h-patches grown on CYE plates, resuspended in 1 ml ice-cold sterile ddH_2_O, and centrifuged for 2 min in Eppendorf tubes. The washing step was repeated three times. The pellet was resuspended in 1 ml ice-cold sterile glycerol, pelleted for 5 min and resuspended in 1 ml ice-cold sterile glycerol, from which 100 μl aliquots were either frozen at -80°C or used for transformation. Electroporation was carried out adding 500 ng DNA and transferring the mixture to a cooled Bio-Rad 0.2-cm cuvette for electroshock with a Bio-Rad GenePulser Xcell set at 2.0 kV, 25 μF, and 200 Ω. After electroporation, 1 ml of AYE broth [1% yeast extract, 1% ACES pH 6.9, 3.3 mM L-cysteine, 0.33 mM Fe(NO_3_)_3_] was added, supplemented with thymidine when required, and the mixture was transferred to a 10 ml tube for incubation for 6 h at 37°C with orbital shaking. The cells were then plated on CYE supplemented with the appropriate antibiotics.

For *B. henselae*, a plate grown for 2 to 3 days was harvested with a sterile cotton swab and resuspended in 950 μl of LB. The suspension was centrifuged at 4,000 rpm for 5 min at 4°C, and the pellet was washed in 950 μl of ice-cold 10% glycerol (three times); 40 μl of these competent cells was transferred to a cooled tube, and 3 μl of DNA (300 ng/μl) was added. The mixture was incubated on ice for 15 min and transferred to a cooled Bio-Rad 0.2-cm cuvette for electroshock with a Bio-Rad Pulse controller II at 2.5 kV/cm, 25 μF, and 200 Ω. After electroporation, 1 ml of SB broth (RPMI 1640 plus L-glutamine, 42 mM HEPES, 1% sodium pyruvate, 5% heat-inactivated fetal calf serum, and 5% sheep blood lysate) was added, and the mixture was transferred to an Eppendorf tube for incubation for 3.5 h at 37°C under 5% CO_2_ conditions with slow shaking. The cells were then centrifuged at 4,000 rpm for 4 min at room temperature. The pellet was resuspended in 40 μl SB broth and plated on CBA supplemented with the appropriate antibiotics.

### Cell Lines and Cell Culture Conditions

The cell lines used for bacterial infections are listed in **Table 4**. EA.hy926 and HeLa cell lines were routinely grown in Dulbecco’s modified Eagle medium (DMEM; Lonza or Gibco), and Chinese Hamster Ovary (CHO) cells were maintained in minimal essential medium MEMα (Gibco); both media were supplemented with 10% heat inactivated fetal bovine serum (FBS; Lonza or Sigma). Cells were incubated at 37°C under 5% CO_2_.

**Table 4:** Mammalian cell lines used in this work.

Name	Description	Reference
CHO FcγRII	Chinese hamster ovary cells producing the FcγRII protein	Joiner et al., 1990
EA.hy926	Fusion cell line of human umbilical vein endothelial cells (HUVEC) and adenocarcinomic human alveolar basal epithelial cells (A549)	ATCC CRL-2922
HeLa	Human epithelial cells of cervix adenocarcinoma	ATCC CCL-2
HeLa 229	Human epithelial cells of cervix adenocarcinoma	ATCC CCL-2.1

### Infections

*Bartonella henselae* strains containing the appropriate plasmids were grown on CBA plates for 3 to 4 days. Human cells were seeded 1 day before infection. For routine infections, cells were seeded in 6-well plates (80,000 cells per well) in 3 ml of medium. When the purpose of the infection was to select human cells that had stably acquired the plasmid transferred from *B. henselae*, infections were performed in 10-cm tissue culture dishes seeded with 450,000 cells in 12 ml of medium. The day of infection, DMEM was replaced by M199 medium (Gibco) supplemented with 10% FBS and appropriate antibiotics to select for the *B. henselae* strains to be added. The bacteria were recovered from the CBA plate and resuspended in 1 ml of PBS. The number of bacteria was calculated considering that an OD_600_ of 1 corresponds to 10^9^ bacteria/ml (Kirby and Nekorchuk, 2002). Bacteria were added to the human cells to get a multiplicity of infection (MOI) of 400 bacteria per host cell. The dishes or plates were incubated for 72 h at 37°C under 5% CO_2_.

*Coxiella burnetii* strains containing the appropriate plasmids were grown for 6 days in liquid cultures. 25,000–50,000 HeLa 229 cells were seeded in DMEM 5% FBS into 24-well plates 6–8 h before they were infected at a MOI of 500, unless specified otherwise. Bacteria were quantified measuring genome equivalents (GE) as previously described (Newton et al., 2014). Infections were incubated for 96 h at 37°C under 5% CO_2_. Wells for quantification of intracellular replication were washed once with PBS at approximately 15 h post infection (hpi) before the addition of fresh DMEM 5% FBS. Wells for flow cytometry experiments were not washed.

*Legionella pneumophila* strains containing the appropriate plasmids were harvested from a heavy patch (after 48 h growth on CYE plates), and used to infect CHO FcγRII cells, stably expressing the receptor FcγRII. This receptor allows *L. pneumophila* opsonized with anti-*Legionella* antibodies to be internalized efficiently by non-phagocytic cells (Arasaki and Roy, 2010). FcγRII cells were grown to near confluency in 24-well dishes. Bacteria were opsonized with rabbit anti-*Legionella* antibody diluted 1/1000 for 20 min at room temperature with shaking. Bacteria were then added to the cells at an estimated MOI of 10. The cells were centrifuged 5 min at 1000 rpm and incubated for 1 h, washed three times with PBS (Gibco) and incubated in fresh media for 24 h at 37°C under 5% CO_2_.

### Detection of GFP Positive Cells by Flow Cytometry

At the indicated hours post infection (hpi) indicated for each bacteria, infected cells were washed with PBS, trypsinized, and analyzed by flow cytometry using a Cytomics FC500 flow cytometer (Beckman Coulter) for *B. henselae* infections, or a BD Accuri C6 flow cytometer (BD Biosciences) for *L. pneumophila* and *C. burnetii* infections. Data were analyzed using the software for each cytometer and FlowJo (Tree Star, Inc.) software. Singlet cells were gated based on SSC-H/FSC-H and GFP positive cells (detected in the FL1-H channel) were gated based on uninfected control cells. The gate was set to approximately 0.05% GFP^+^ cells in the uninfected control sample.

### Fluorescence Microscopy

At the indicated hpi, wells with infected cells were washed with PBS and the plates were placed directly on a Nikon Eclipse TE2000-S inverted fluorescence microscope with a 10× objective lens. Digital images were acquired with a microscope camera (Photometrics CoolSNAP EZ) controlled by SlideBook^TM^ (Intelligent Imaging Innovations).

### Detection of Stable Integrants

At 72 hpi, either 500 μg/ml G418 disulfate salt (Sigma–Aldrich) or 300 μg/ml Hygromycin B (Invitrogen), as appropriate, were added to HeLa cells infected with *B. henselae*, and selection was maintained for 4 to 5 weeks. Resistant colonies on the plates were counted.

In order to calculate the integration rate, integration experiments were always performed in parallel with infections to measure GFP positive cells by flow cytometry. The resulting percentage of GFP positive cells was extrapolated to the number of cells in the 10-cm plate used to detect integrants, and the number of resistant colonies was divided by the inferred number of GFP positive cells.

### Determination of Genome Equivalents (GE)

Quantification of *C. burnetii* intracellular replication was performed as described in Newton et al. (2014). Briefly, infected HeLa cells were lysed in ddH_2_O at specific time points post infection. Total genomic DNA was extracted using the Illustra Bacteria GenomicPrep Mini Spin Kit (GE Healthcare) and GE were quantified by qPCR using *dotA*-specific primers (GCGCAATACGCTCAATCACA, CCATGGCCCCAATTCTCTT). The generation of this short PCR product is not affected by the presence of a transposon in the *dotA*::Tn mutant strain.

## Results

The conjugative relaxase TrwC can be translocated through the T4SS VirB/D4 of *B. henselae* to human cells, where it promotes the integration of the transferred DNA into the recipient genome (Gonzalez-Prieto et al., 2017). In this work, we wanted to test whether this is a unique feature of TrwC and VirB/D4, or other systems can also be combined to deliver and integrate DNA into human cells.

To test DNA transfer mediated by the relaxase MobA of the mobilizable plasmid RSF1010, we constructed a derivative carrying an eukaryotic eGFP expression cassette to detect gene expression from the human cell nucleus. An insertion of an ampicillin resistance cassette in *mobA* served as a negative control. The insertion is located in the 5′ region of the ORF, thus not affecting the expression of the ORFs *mobB* and especially *repB’*, which encodes a DNA primase required for plasmid replication. We observed that this *mobA^-^* construct had a higher copy number than the parental plasmid, as judged from the amount of DNA extracted from parallel cultures (data not shown). This phenomenon has previously been reported, and attributed to the repressor role of MobA/RepB in replication (Frey et al., 1992).

**FIGURE 1:**
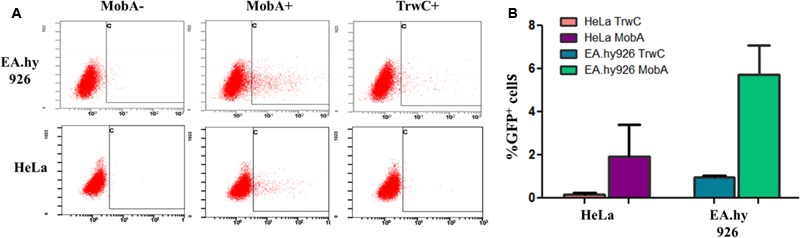
Transient expression of transferred DNA in HeLa and EA.hy926 cells. Pools of cells obtained at 3 days post infection with *B. henselae* were analyzed by flow cytometry using uninfected cells as control. **(A)** Representative plots (cell granularity versus GFP intensity). The square marks the population considered as positive. The relaxase present in each experiment is indicated on top of the panels. **(B)** Mean values of the percentage of GFP positive cells from 2 to 4 independent experiments, after subtracting the background values of the negative controls with no relaxase. The relaxase present in each experiment is indicated.

These plasmids (pAA58 and pMTX808; **Table 2**) were introduced in *B. henselae*, and the resulting strains were used to infect both EA.hy926 and HeLa human cell lines. The former is derived from HUVEC cells, which are the natural target of *B. henselae in vivo*; however, HeLa cells can also be infected by *B. henselae* with lower efficiency, and we showed that TrwC-mediated DNA transfer takes place to HeLa cells as well (Gonzalez-Prieto et al., 2017). *B. henselae* carrying plasmids coding for either MobA or TrwC, or relaxase mutants as negative controls, were used for infections. To assess transfer of the plasmid DNA to the human cells, flow cytometry was used to quantify the expression of the eGFP cassette per cell, thus allowing the determination of the percentage of GFP positive cells. The results are shown in **Figure 1** and **Table 5**, top 8 rows. We observed DNA transfer when the plasmids encoded a functional relaxase, and background levels in the absence of a relaxase. DNA transfer rates were notably higher when using MobA as the leading relaxase compared to TrwC.

**Table 5:** Rates of DNA transfer to mammalian cells through T4ASS and T4BSS.

Donor bacteria (genotype)	T4SS	Transfer system	Relaxase	Infected cells	GFP^+^ mammalian cells^(1)^	
					Flow cyt %	Scope
*Bh* RSE247 (wt)	Functional	RSF1010	MobA	EA.hy926	5.72 ± 1.37	nq^(2)^
*Bh* RSE247 (wt)	Functional	RSF1010	–	EA.hy926	0.29 ± 0.07	nq^(2)^
*Bh* RSE247 (wt)	Functional	R388	TrwC	EA.hy926	1.00 ± 0.09	nq^(2)^
*Bh* RSE247 (wt)	Functional	R388	–	EA.hy926	0.14 ± 0.19	nq^(2)^
*Bh* RSE247 (wt)	Functional	RSF1010	MobA	HeLa	2.00 ± 1.48	nq^(2)^
*Bh* RSE247 (wt)	Functional	RSF1010	–	HeLa	0.07 ± 0.05	nq^(2)^
*Bh* RSE247 (wt)	Functional	R388	TrwC	HeLa	0.20 ± 0.03	nq^(2)^
*Bh* RSE247 (wt)	Functional	R388	–	HeLa	0.04 ± 0.06	nq^(2)^
*Lp* Lp02 (wt)	Functional	RSF1010	MobA	CHO FcγRII	0.35 ± 0.12	nq^(2)^
*Lp* Lp03 *(dotA)*	No transport	RSF1010	MobA	CHO FcγRII	0.03 ± 0.05	<5 × 10^-6^
*Lp* Lp02 (wt)	Functional	RSF1010	–	CHO FcγRII	0.00 ± 0.00	<5 × 10^-6^
*Lp* CR503 (*icmS icmW)*	No chaperone	RSF1010	MobA	CHO FcγRII	0.00 ± 0.00	<5 × 10^-6^
*Lp* Lp02 (wt)	Functional	R388	TrwC	CHO FcγRII	0.00 ± 0.00	<5 × 10^-6^
*Lp* Lp02 (wt)	Functional	R388	TrwC-RalF	CHO FcγRII	0.00 ± 0.00	1 × 10^-5^
*Lp* Lp03 *(dotA)*	No transport	R388	TrwC-RalF	CHO FcγRII	nq^(2)^	<5 × 10^-6^
*Lp* Lp02 (wt)	Functional	R388	–	CHO FcγRII	nq^(2)^	<5 × 10^-6^
*Lp* CR503 (*icmS icmW)*	No chaperone	R388	TrwC-RalF	CHO FcγRII	nq^(2)^	2 × 10^-5^
*Cb* intergenic::Tn (wt)	Functional	RSF1010	MobA	HeLa	0.56 ± 0.53	nq^(2)^
*Cb dotA*::Tn	No transport	RSF1010	MobA	HeLa	0.04 ± 0.02	nq^(2)^
*Cb* intergenic::Tn (wt)	Functional	RSF1010	–	HeLa	0.10^(3)^ ± 0.04	<5 × 10^-6^

***^(1)^**DNA transfer is measured as the ratio of mammalian recipient cells expressing GFP. Data from flow cytometry (left column) show the percentage of GFP positive cells (mean ± SD of two to eight independent assays). Infected cells were also screened visually under the microscope (right column). Positive cells were counted and divided by the total number of cells per well (estimated as 200.000). The screen was performed at least twice for each condition. **^(2)^**nq, not quantified. **^(3)^**Due to higher background (see text for details).*

In order to measure genomic integration of the transferred DNA, we constructed plasmid derivatives encoding antibiotic resistance cassettes (see **Table 2**). The plasmids containing R388 conjugative elements carried a neomycin gene; however, this was not used in these experiment because of the presence of a kanamycin resistance gene in the RSF1010K backbone, which could lead to recombination between both cassettes. Instead, a hygromycin resistance cassette was inserted. In order to avoid an effect caused by the different antibiotic selections applied, we also constructed Hygromycin-resistant derivatives encoding TrwC (**Table 2**), and we found that TrwC-mediated integration rate did not vary when the selection applied was hygromycin B or Geneticin (data not shown).

**FIGURE 2:**
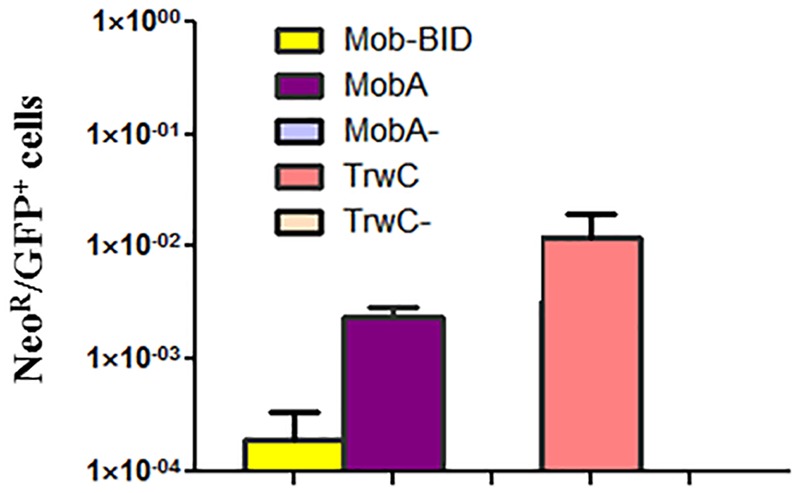
DNA integration rate for different relaxases. HeLa cells were infected with *B. henselae* containing the plasmids encoding the indicated relaxases (or the negative controls lacking the relaxase) and at 3 days post infection, cells were either analyzed by flow cytometry (to determine transient expression of eGFP), or subjected to antibiotic selection (to determine permanent expression of the antibiotic resistance gene). The graph shows the ratio between the number of antibiotic-resistant cells and the number of GFP positive cells.

HeLa cells were used as target cells to measure DNA integration, because in contrast to EA.hy926 cells HeLa cells show enhanced survival during the 4–5 weeks of antibiotic selection required to measure resistant colonies (Gonzalez-Prieto et al., 2017). The cells were infected with *B. henselae* carrying the different plasmids. A plasmid derived from the cryptic *Bartonella* plasmid pBGR1 was also assayed for comparison, since it has been reported that its relaxase mediates DNA transfer but does not promote integration of the transferred DNA (Gonzalez-Prieto et al., 2017). After applying the antibiotic selection, resistant colonies were counted, and integration rates were calculated dividing this number by the number of GFP positive cells determined in parallel infection experiments (see Materials and Methods for details). The results (**Figure 2**) indicate that the integration rate for the MobA constructs was approximately one-log higher than in case of Mob-BID, which suggest that MobA promotes integration of the transferred DNA. It can also be observed that TrwC has a stronger effect on integration than MobA (approximately five-fold higher DNA integration).

**FIGURE 3:**
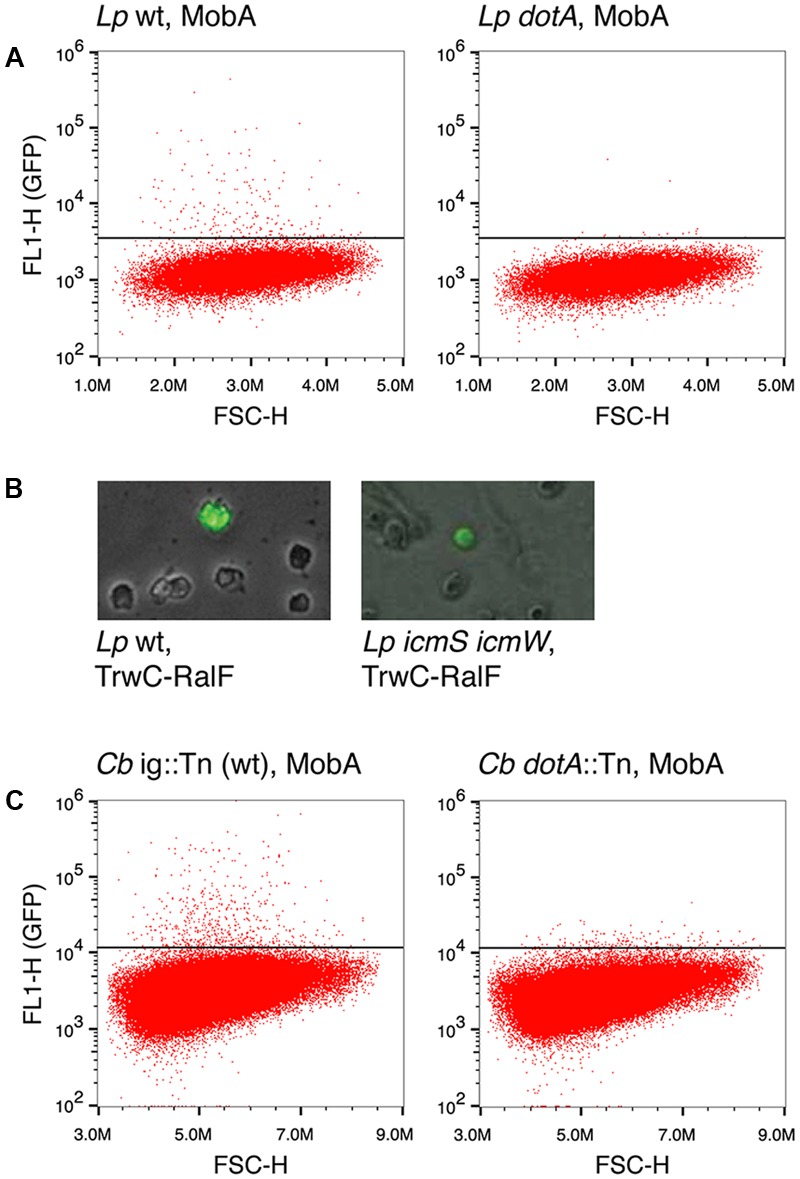
DNA transfer through the Dot/Icm T4BSS. **(A)** Representative flow cytometry plots for quantification of GFP positive CHO FcγRII cells after infection with *L. pneumophila* strains Lp02 (wild type, left panel) or Lp03 (*dotA* mutant, right panel) harboring plasmid pAA58, which encodes MobA. **(B)** Fluorescence microscope images showing GFP positive CHO FcγRII cells infected with *L. pneumophila* strains Lp02 (wild type, left panel) or CR503 (*ΔicmSΔicmW* mutant, right panel) harboring plasmid pAA12, which encodes TrwC-RalF. **(C)** Representative flow cytometry plots for quantification of GFP positive HeLa cells after infection with *C. burnetii* strains RSA439 intergenic::Tn (wild type, left panel) or RSA439 *dotA*::Tn (*dotA* mutant, right panel) harboring plasmid pAA58, which encodes MobA. ig, intergenic.

Earlier studies reported Dot/Icm-dependent conjugative DNA transfer of RSF1010 (Vogel et al., 1998), implying that MobA can mediate the translocation of an attached DNA substrate through the T4BSS Dot/Icm of *L. pneumophila*. Thus, we asked whether the Dot/Icm T4SS could also promote DNA transfer to mammalian cells upon infection by *L. pneumophila*. In addition to testing MobA-mediated transfer, we tested DNA transfer mediated by TrwC and TrwC-RalF, a fusion protein carrying the C-terminal 20 residues of the *L. pneumophila* Dot/Icm substrate RalF, that has been shown to be sufficient for translocation (Nagai et al., 2005). In contrast to the infection experiments done with *B. henselae*, for infections with *L. pneumophila* a MOI of 10 was used and DNA transfer was monitored at 24 hpi. As shown in **Figure 3A** and **Table 5**, we detected GFP positive cells after infection by a mechanism dependent on the Dot/Icm T4BSS and the relaxase MobA. Thus, we show for the first time that DNA transfer can occur through a T4BSS into mammalian cells. Using the same flow cytometry assay, we did not detect GFP positive cells above the background when the mobilizable plasmids encoded the relaxase TrwC or TrwC-RalF. However, inspection of the infected cells by fluorescence microscopy did reveal a small number of positive cells that expressed GFP uniformly and strongly after infection with *L. pneumophila* producing TrwC-RalF (**Figure 3B**). Positive cells were not observed in the negative controls or with TrwC-encoding plasmids.

The rate of DNA transfer was highly dependent on the conjugative DNA processing system used. This could be due to different relaxase recruitment efficiencies. The Dot/Icm T4BSS recruits a subset of its substrates through a chaperone complex formed by IcmS and IcmW (Cambronne and Roy, 2007). To determine if recruitment of the relaxases was dependent on this complex, a *L. pneumophila ΔicmS ΔicmW* mutant strain was used in infection experiments carrying plasmids which encode either MobA or TrwC-RalF. The results (**Table 5** and **Figure 3B**) indicate that the absence of IcmSW did not affect DNA transfer mediated by TrwC-RalF, while DNA transfer mediated by MobA was abolished in the absence of IcmSW.

The Dot/Icm T4BSS of *L. pneumophila* is closely related to that of *C. burnetii*, and several reports have shown that both can recruit the same effector proteins and cross-complement *icmSW* mutants (Zamboni et al., 2003; Zusman et al., 2003; Carey et al., 2011). Thus, we decided to test MobA-mediated DNA transfer through the Dot/Icm T4BSS of *C. burnetii*. HeLa cells were infected with *C. burnetii* strains harboring the plasmids with and without MobA at a MOI of 500, and GFP expression was investigated at 4 days post infection. The results are shown in **Table 5**, and **Figure 3C** shows representative plots. Similar to what was observed with *L. pneumophila*, GFP positive cells were only detected when the Dot/Icm T4BSS and the MobA relaxase were present.

**FIGURE 4:**
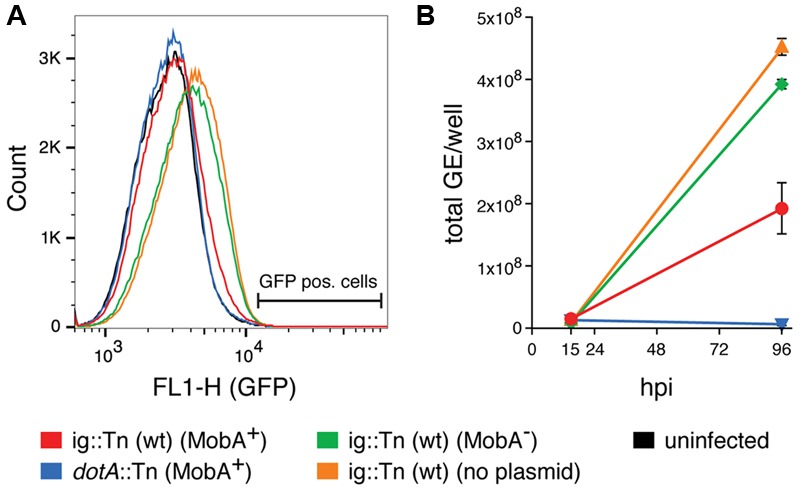
The presence of MobA interferes with intracellular replication of *C. burnetii* in HeLa cells. **(A)** Flow cytometry histogram analysis of HeLa cells infected with the indicated *C. burnetii* strains at 4 days post infection. The location of GFP positive cells (see **Figure 3C** and Materials and Methods) is indicated. **(B)** Quantification of intracellular replication of *C. burnetii*. Total genome equivalents (GE)/well are shown at two time points post infection for infections with the indicated strains at a MOI of 50. The bottom strain legend applies for **(A,B)**. The presence or absence of plasmid-encoded MobA is indicated in brackets. ig, intergenic. hpi, hours post infection.

Performing these experiments, we observed a difference in the background fluorescence intensity of HeLa cells depending on the bacterial strain used for infection. A representative flow cytometry histogram is shown in **Figure 4A**. The background GFP fluorescence peak shifts toward a higher intensity when HeLa cells were infected with wild type *C. burnetii* or wild type *C. burnetii* harboring the plasmid with the *mobA* mutation, but not when cells were infected with wild type *C. burnetii* carrying the plasmid with the intact *mobA* gene. This higher fluorescence did not correspond to DNA transfer, since we did not detect any proper GFP positive cells by flow cytometry or using microscopy, but it contributed to a minimal raise in the background frequencies observed when infecting with a *mobA^-^* strain (see **Table 5**). However, the difference in background fluorescence may be attributed to a different amount of intracellular bacteria per cell. To test this hypothesis, HeLa cells were infected at a MOI of 50 and the number of intracellular *C. burnetii* was determined by measuring GE at two time points post infection. The results are shown in **Figure 4B**. A strain carrying the *mobA*-deficient plasmid replicates nearly as efficiently as a strain with no plasmid. In contrast, the same strain carrying a plasmid that encodes a functional MobA protein was severely impaired in intracellular replication. A *dotA* mutant that fails to replicate intracellularly due to the absence of a functional T4SS was used as a control in this assay.

## Discussion

In our previous reports, we showed that the conjugative relaxase TrwC can be translocated to human cells through the T4SS VirB/D4 of *B. henselae* (Fernandez-Gonzalez et al., 2011), and also that it promotes integration of the transferred DNA into the recipient genome (Gonzalez-Prieto et al., 2017). Whether these abilities were unique for TrwC and VirB/D4 remained to be tested. In this work, we report that different relaxases and T4SS can be used to transfer DNA to human cells and to promote DNA integration. In other words, relaxases and T4SS from various bacterial species can be combined to create tools intended to genetically modify specific human target cells in a permanent way, thus generating enormous biotechnological potential.

Firstly, we compared the ability of different relaxases to transfer DNA to mammalian cells and to promote DNA integration into the recipient genome when translocated by the same T4SS, VirB/D4. Human cells were infected with *B. henselae* carrying derivatives of the mobilizable plasmid RSF1010, encoding the relaxase MobA; with constructs containing the conjugative processing elements of the self-transferable plasmid R388, which encodes the relaxase TrwC; or with derivatives of *B. henselae* cryptic plasmid pBGR1, coding for the relaxase Mob fused to the BID signal for efficient recruitment by VirB/D4 (Schroder et al., 2011). When the three plasmids are compared in terms of DNA transfer and integration rates (**Figures 1, 2**), we find that these vary significantly, with RSF1010 being the most efficiently transferred, while TrwC is the relaxase showing higher integration rates. The rate of DNA transfer is probably proportional to the efficiency with which the relaxase is recruited to the T4SS machinery; this assumption comes from previous works showing that the relaxase Mob itself could transfer DNA to human cells with barely detectable frequency, but when a recruitment secretion signal was fused to its C-terminal end, it transferred DNA to similar frequencies than TrwC (Schroder et al., 2011). In addition, in case of R388, a deletion of the conjugative coupling protein, a component believed to play a key role in the recruitment of the conjugative substrate, caused DNA transfer rates to drop 10-fold (Fernandez-Gonzalez et al., 2011). The relaxase MobA belongs to a mobilizable plasmid which hijacks the T4SS of co-residing conjugative plasmids, so it can be translocated through various T4SS; thus, it is plausible that the requirements for MobA recruitment are less stringent. In fact, the C-terminal 48 residues of MobA were shown to direct translocation of a Cre fusion through the VirB T4SS of *A. tumefaciens* into plant cells (Vergunst et al., 2005). Now, we show that MobA can also be translocated through a T4ASS into mammalian cells.

The ability to enhance integration of the transferred DNA into the recipient cell genome must reside in an intrinsic property of the relaxase, which is the only protein entering the recipient cell covalently attached to the transferred DNA strand. We report here that the promiscuous relaxase MobA also promotes DNA integration, resulting in resistant colonies with about 10-fold higher frequency than Mob-BID, which does not promote integration above background levels obtained by DNA transfection (Gonzalez-Prieto et al., 2017), but roughly five-fold lower frequency than TrwC. These differences observed among relaxases could be due to differential nuclear targeting, catalytic activity, or binding affinity to its target, which could protect the DNA ends, thus favoring integration by host-mediated mechanisms, as previously suggested (Gonzalez-Prieto et al., 2017). Subcellular localization of TrwC and MobA in human cells showed no preferential nuclear localization for either relaxase (Silby et al., 2007; Agundez et al., 2011). It is noteworthy that TrwC catalyzes site-specific recombination on supercoiled DNA substrates (Cesar et al., 2006), while MobA was shown to catalyze site-specific recombination between two *oriT* copies when the substrate was single-stranded (Meyer, 1989), and other relaxases do not catalyze this reaction at all. Although the integration pattern in the human genome is random (Gonzalez-Prieto et al., 2017), site-specific recombination ability could play a role in strand-transfer reactions when the nucleoprotein complex is directed to a nicked DNA strand by the host repair machinery.

MobA can be translocated by the T4BSS of *L. pneumophila*, alone or bound to DNA, into recipient bacteria (Vogel et al., 1998; Luo and Isberg, 2004). These results prompted us to test its translocation by T4BSS into mammalian cells. Our results (**Figure 3**) show for the first time that DNA transfer to human cells can also be accomplished through the Dot/Icm T4BSS of *L. pneumophila* and *C. burnetii*, only remotely related to T4ASS. Thus, it is reasonable to assume that DNA translocation may be an intrinsic ability of T4SS. An important difference between both Dot/Icm systems is the temporal pattern of secretion: while *L. pneumophila* has been shown to secrete effectors as internalization into host cells is initiated (Nagai et al., 2005) in case of *C. burnetii* effector translocation is initiated when the pathogen has reached an acidified lysosomal compartment (Newton et al., 2013); thus, DNA transfer in *C. burnetii* must occur from within the *Coxiella*-containing vacuole.

DNA transfer was dependent on the presence of the Dot/Icm T4SS and a functional relaxase, as expected for a *bona fide* conjugation-like DNA transfer process. The wide differences in DNA transfer rates depending on the relaxase (MobA, TrwC, or TrwC-RalF, including the translocation signal of the natural T4SS substrate RalF) and on the presence/absence of the chaperones IcmSW (see **Table 5**) support the concept that relaxase recruitment is the main driver of DNA transfer.

During the course of performing *C. burnetii* infection experiments, we noticed an inhibition of *C. burnetii* intracellular replication caused by the presence of RSF1010 derivatives carrying a functional MobA relaxase while isogenic strains with a *mobA* mutation did not affect growth (**Figure 4**). Similarly, RSF1010 conjugation was shown to inhibit intracellular replication and virulence of *L. pneumophila* (Segal and Shuman, 1998), probably by MobA interference with effector secretion by Dot/Icm. This result should be taken into account when using vectors based on RSF1010, which are the more commonly used by both *L. pneumophila* and *C. burnetii*.

Finally, an attractive question that remains open is the possible biological role, if any, of DNA transfer to mammalian cells by bacterial pathogens harboring a T4SS. Is the DNA transfer ability an evolutionary remnant of the conjugative T4SS from which the T4SS involved in virulence probably have evolved? Or is it an ability which the pathogens have evolved to use to their own benefit, in the same way as *A. tumefaciens* uses it to subvert its eukaryotic host cell?

**FIGURE 5:**
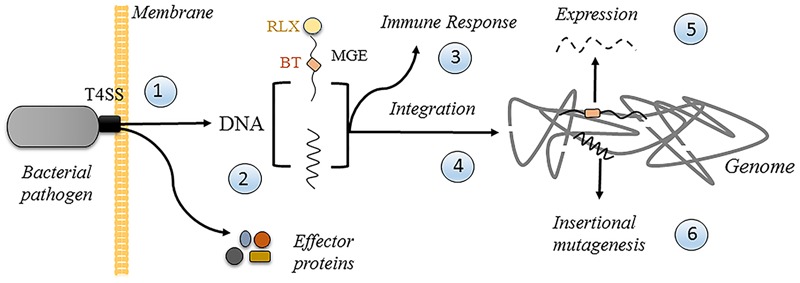
Possible fates of translocated DNA in a human cell. See text for details. Random DNA is represented by the jagged line. Mobile genetic element (MGE) is represented by the wavy line. RLX, relaxase (small yellow sphere). BT, beneficial trait (small orange box).

In support of the first possibility, it is relevant to point out that in spite of many attempts, no T4 protein, protein domain or amino acid residue has been identified to date, which is specifically involved in DNA transfer. All analyzed mutants in T4 components, even in the conjugative coupling protein ATPase, affected DNA and protein translocation to the same extent, leading to the suggestion that relaxase and DNA translocation may have the same molecular requirements (de Paz et al., 2010; Larrea et al., 2017). Thus, the ability to transfer DNA could not be lost in a T4SS even if it evolved to only secrete proteins. However, the potential of DNA transfer for long-term subversion of the host cells makes it attractive to think that pathogens may utilize such a process for their own profit. **Figure 5** illustrates the possible fates of secreted DNA in a human cell. A pathogen translocates effector proteins and DNA through its T4SS once in contact with the membrane (1 in **Figure 5**), whether it is from within a vacuolar compartment, as in case of *C. burnetii*, or from the outside. The secreted DNA could either be random DNA, as proposed for *H. pylori* (Varga et al., 2016), or a specifically recruited mobile genetic element (MGE), in which case a dedicated transfer system would attach a relaxase to its end (2). The cytoplasmic DNA could elicit an immune response (3), as proposed for *H. pylori* (Varga et al., 2016), which could be used by the pathogen for its own benefit. DNA could also get integrated into the host cell genome (4) by the host repair/recombination systems, and/or by the covalently attached conjugative relaxase. Integration will lead to the stable expression of the encoded information (5), including any beneficial traits that the pathogen may have evolved to encode in MGE for that purpose. Finally, random integration has an inherent risk of insertional mutagenesis (6), which could lead to increased growth of the host cell, thereby promoting the extension of the niche of the pathogen.

In this context, it has to be stressed that human pathogens contain many poorly characterized MGE, which could be substrates for DNA transfer (in addition to the possibility of sporadic transfer of visiting promiscuous plasmids, such as RSF1010). As examples from the pathogens used in this study, the pBRG1 cryptic plasmid of *B. henselae* can be recruited by VirB/D4 and translocated to human cells (Schroder et al., 2011); conjugative transfer of chromosomal DNA has been reported for *L. pneumophila* (Miyamoto et al., 2003), and its genome includes several genomic islands; and notably, a cryptic plasmid in *C. burnetii* is enriched in important effector genes (Voth et al., 2011); it is tempting to speculate that this plasmid may be transferred to the host cell.

## Author Contributions

All authors contributed to the conception and design of the work, data acquisition and/or analysis. All authors contributed to drafting, revising, and final approval of the work. All authors agree to be accountable for all aspects of the work.

## Conflict of Interest Statement

The authors declare that the research was conducted in the absence of any commercial or financial relationships that could be construed as a potential conflict of interest. The reviewer JRM and handling Editor declared their shared affiliation, and the handling Editor states that the process nevertheless met the standards of a fair and objective review.
